# Long-Term Safety of Topical Bacteriophage Application to the Frontal Sinus Region

**DOI:** 10.3389/fcimb.2017.00049

**Published:** 2017-02-24

**Authors:** Amanda J. Drilling, Mian L. Ooi, Dijana Miljkovic, Craig James, Peter Speck, Sarah Vreugde, Jason Clark, Peter-John Wormald

**Affiliations:** ^1^Department of Surgery-Otolaryngology Head and Neck Surgery, The University of AdelaideAdelaide, SA, Australia; ^2^Adelaide Pathology PartnersAdelaide, SA, Australia; ^3^School of Biological Sciences, Flinders UniversityBedford Park, SA, Australia; ^4^Fixed Phage LimitedGlasgow, UK

**Keywords:** bacteriophage, therapeutic safety, topical, inflammation, cilia, *Staphylococcus aureus*

## Abstract

**Background:** *Staphylococcus aureus* biofilms contribute negatively to a number of chronic conditions, including chronic rhinosinusitis (CRS). With the inherent tolerance of biofilm-bound bacteria to antibiotics and the global problem of bacterial antibiotic resistance, the need to develop novel therapeutics is paramount. Phage therapy has previously shown promise in treating sinonasal *S. aureus* biofilms.

**Methods:** This study investigates the long term (20 days) safety of topical sinonasal flushes with bacteriophage suspensions. The bacteriophage cocktail NOV012 against *S. aureus* selected for this work contains two highly characterized and different phages, P68 and K710. Host range was assessed against *S. aureus* strains isolated from CRS patients using agar spot tests. NOV012 was applied topically to the frontal sinus region of sheep, twice daily for 20 days. General sheep wellbeing, mucosal structural changes and inflammatory load were assessed to determine safety of NOV012 application.

**Results:** NOV012 could lyse 52/61 (85%) of a panel of locally derived CRS clinical isolates. Application of NOV012 to the frontal sinuses of sheep for 20 days was found to be safe, with no observed inflammatory infiltration or tissue damage within the sinus mucosa.

**Conclusion:** NOV012 cocktail appears safe to apply for extended periods to sheep sinuses and it could infect and lyse a wide range of *S. aureus* CRS clinical isolates. This indicates that phage therapy has strong potential as a treatment for chronic bacterial rhinosinusitis.

## Introduction

*Staphylococcus aureus* is an opportunistic bacterial pathogen forming biofilms, which are known to be involved in a number of infective chronic diseases (Petti and Fowler, 2003; Ziran, 2007; Baldoni et al., 2009; Foreman and Wormald, 2010; Singhal et al., 2010, 2011; Jervis-Bardy and Wormald, 2012). These include osteomyelitis (Ziran, 2007), endocarditis (Petti and Fowler, 2003), as well as infections of indwelling devices (Baldoni et al., 2009). In addition, sinonasal bound biofilms of *S. aureus* are known to impact negatively in chronic rhinosinusitis (CRS). The presence of such infections in CRS reportedly leads to more frequent out-patient visits (Singhal et al., 2011), increased risk of recurrent infections and antibiotic use (Jervis-Bardy and Wormald, 2012) as well as poorer post-operative progression (Foreman and Wormald, 2010; Singhal et al., 2010). Such biofilms are up to 1000-fold more tolerant of current antibiotic therapies than their planktonic counterparts (Anwar et al., 1990). Further, increased levels of antibiotic resistance observed in pathogenic bacteria around the globe (Roca et al., 2015) also limit the success of antibiotic therapies. It is important that new therapies which effectively treat such infections are identified.

One alternative to antibiotics, originally described in the early 1900s and coming back into focus, is bacteriophage “phage” therapy (Carlton, 1999). Beneficially, phages are not only effective against planktonic infections, but can also infect and lyse biofilm-bound cells (Doolittle et al., 1995; Corbin et al., 2001; Tait et al., 2002; Sillankorva et al., 2004; Curtin and Donlan, 2006; Cerca et al., 2007; Lu and Collins, 2007; Carson et al., 2010; Fu et al., 2010). Recently, we have shown that topical phage therapy has potential against pathogenic *S. aureus* bacterial biofilms, using an animal model of rhinosinusitis (Drilling et al., 2014a). Our previous work demonstrated the safety of once-daily phage application into the frontal sinuses of sheep for 3 days (Drilling et al., 2014a). The first aim of the current work was to examine the safety of phage administration for a substantially longer period of time. In the current study we investigated the effects of a longer-term (20 day) application of twice-daily frontal sinus bacteriophage flushes in sheep. A cocktail of two phages against *S. aureus*, designated NOV012, was used. NOV012 contains two highly-characterized phages, K710 and P68. Both the parental version of phage K710, phage K (O'Flaherty et al., 2004), and phage P68 (Vybiral et al., 2003) have had their genomes completely sequenced, and they have been shown to lack any known genes that could increase the virulence of *S. aureus* or confer resistance to antibiotics (Vybiral et al., 2003; O'Flaherty et al., 2004). Phages K710 and P68 have been shown to be active against a wide range of *S. aureus* isolates from the United Kingdom and Europe (Takac and Blasi, 2005; J Clark, personal communication). The second aim of this work was to extend these observations by examining the susceptibility of local (Australian) *S. aureus* isolates to the phages in NOV012.

## Methods

### Bacteriophage

A phage cocktail (NOV012) comprised of preparations of highly purified phages, K710 and P68, was obtained from Novolytics Pty. Ltd. (Warrington, United Kingdom). This cocktail of phages is functionally similar to the CTSA cocktail (Special Phage Services, Brookvale, NSW, Australia) which we used previously (Drilling et al., 2014a). For commercial reasons CTSA was unavailable. The concentration of phages in NOV012 stock was maintained at 1 × 10^8^ PFU/mL for *in vivo* and *in vitro* work. To produce a heat-inactivated version of the cocktail, the phage stock was treated at 121°C for 15 min and tested by plaque assay to confirm complete inactivation prior to use.

### Bacteria

Investigation of *S. aureus* strains isolated from CRS patients was approved by The Queen Elizabeth Hospital Human Ethics Committee. Written informed consent was obtained for all study participants. To isolate *S. aureus* from sinonasal swabs, Columbian blood agar plates, colistin nalidixic acid plates or cystine lactose electrolyte deficient plates (all from Thermofisher Scientific, Australia) were employed. Latex agglutination tests and antibiotic sensitivity testing were performed by the commercial laboratory Adelaide Pathology Partners and used to confirm identification of *S. aureus* and MRSA strains (data not shown). Antibiotic resistance was determined using disc diffusion methods as according to Clinical and Laboratory Standards Institute (CLSI) recommendations (CLSI, 2012). Isolates were then subcultured in nutrient broth overnight (Thermo Fisher, Scoresby, Victoria, Australia) and stored in nutrient broth with 20% glycerol at −80°C.

### CRS bacterial isolate susceptibility to phage infection

Bacterial sensitivity to phage infection was assessed by spotting phage onto bacterial lawns in an agar overlay system. Briefly, *S. aureus* isolates were cultured overnight at 37°C with shaking for 16–18 h in nutrient broth. Overnight cultures were diluted 1:30 in liquid 0.4% nutrient agar which was overlayed onto 1% nutrient agar. Dilutions of P68, K710, and the cocktail of these (NOV012) were spotted onto soft agar plates and allowed to dry. Plates were inverted and incubated overnight at 37°C. Spots were assessed the next day and phage titres and plaque morphologies recorded. Plaques were ranked “+++” (highly sensitive, clear plaque) through to “+” (slightly sensitive, plaque barely discernible), and plaques of status intermediate between these were ranked “++” (moderately sensitive). Results were termed “lysis from without” (LWO) when zones of inhibition were observed however discreet plaques were not evident when the assay used diluted phage stocks. Bacterial isolates were considered susceptible to phages if a plaque was discernible. To determine the strain type of the isolates, pulsed-field gel electrophoresis was used as described (O'Brien et al., 2006). Isolates with ≥80% similarity or <6 band differences were considered the same strain. Efficiency of plating was determined using the concentration of phage determined from infection of the *S. aureus* reference strain ATCC 25923 (Drilling et al., 2014a,b, 2016).

### Frontal sinus access and treatment

Animal work performed in this study was approved by the South Australian Health and Medical Research Institute and the University of Adelaide Animal Ethics Committees. Access to the ovine frontal sinus was achieved through the placement of mini-trephines, as described (Ha et al., 2007). Once accessed, sinuses where flushed twice daily with 50 mL of treatment using an extension cannula for 20 days. Sheep were treated with one of three different treatments: 0.9% saline (“CONT”), 0.9% saline containing 2 × 10^6^ pfu/mL heat-inactivated phage NOV012 (HI*p*) or 0.9% saline containing 2 × 10^6^ pfu/mL active NOV012 (NOV012). Each treatment group consisted of 4 sheep. One trephine from each treatment group became blocked during the treatment, resulting in *n* = 7 sinuses per group. Sheep were monitored for general wellbeing during treatment. At the completion of treatment, sheep were euthanized and sinus tissue harvested for analysis. Microbiology swabs were taken to determine the bacterial composition of the sinus.

### Histology and scanning electron microscopy

Mucosal sections were dissected and placed in either 10% formalin for histological analysis, or scanning electron microscopy (SEM) buffer [4% paraformaldehyde/1.25% glutaraldehyde in phosphate-buffered solution (PBS) with 4% sucrose] for SEM. For histological analysis, tissue was embedded into paraffin blocks, sectioned, mounted on slides, and stained using haematoxylin and eosin (H&E). Sections were examined by an experienced tissue pathologist (author CJ). Sections were identified only by a code to ensure the examining pathologist was unaware of which treatment had been provided to the animals. The examining pathologist graded the tissue for levels of inflammation, oedema, fibrosis, and presence or absence of goblet cell hyperplasia. For SEM, tissue was counterstained using 2% osmium tetroxide and dehydrated using a graded series of 70–100% ethanol washes. The tissue was chemically dried using hexamethyldisilazane (Sigma Aldrich) and mounted on SEM stub specimen mounts (Ted Pella, Redding, CA). The stubs were then coated in carbon using a standard carbon coater (Ted Pella) and viewed using an XL30 field emission Gun scanning electron microscope (Philips, Eindhoven, Netherlands). Five images of each tissue section were captured at magnification 2500× where at least 50% of the image allowed visualization of the tissue surface. Each image was broken down into 2 cm^2^ grid sections and scored either: 1, full cilia coverage; 0.5, some cilia coverage; 0, no cilia present. Not counted, mucus covering cilia or epithelial layer, so tissue could not be visualized.

### Isolation of phage from serum samples

Serum samples were collected from all sheep prior to the first treatment flush. Further, sheep in the inactivated phage group and in the control group had serum samples taken on days 7, 14 and 19 after the first flush. Phage-treated sheep had serum samples taken 10 min, and 1, 2, and 4 h after flush 1, and 18 h after the second flush. Serum samples were not found to contain phages at any of the tested time points. Therefore on days 7, 14, and 19, serum samples were harvested directly after phage flush 1 as well as 1 and 2 h post-flush 1 and 18 h post-flush 2. A mucosal sample (1 g) harvested from each phage-treated sheep on day 21 was processed and filtered as previously described (Drilling et al., 2014a). All harvested serum and mucosal samples were tested for infectious phage using the agar overlay plaque assay method. Serum (1 ml) or processed mucosal sample was mixed with cultures of *S. aureus* strain ATCC 25923 (cultured as above) and incubated at room temperature for 15 min. Samples were then mixed with 2 mL of 0.7% nutrient agar and overlayed onto 1% nutrient agar plates. Plates were examined every 24 h for 3 days for the presence of plaques. Agar overlays were performed in triplicate.

### Statistics

Statistical analyses were performed using SPSS version 23 software (IBM® SPSS® Statistics, New York, USA). Fisher's exact tests were used to compare the range of *S. aureus* stains that the phage preparations (P68 vs. K710 vs. cocktail) could infect and kill. All other statistical comparisons were performed using Kruskal-Wallis analysis and post-hoc with Bonferroni correction (Theodorsson-Norheim, 1986).

## Results

### Infection range of phages P68 and K710 against CRS-derived *S. aureus* clinical isolates

*S. aureus* isolates from 61 patients diagnosed with CRS were examined (Table 1). When tested for strain type by pulse-field gel electrophoresis, 25 different strains were identified. Each strain contained 1–6 different clonal types. Clonal types R1, R3, and T3 were observed in the isolate population more twice. When tested for susceptibility to K710, P68, and NOV012, 36/61 isolates (59%) were found sensitive to phage K710, 45 (74%) were sensitive to P68, and 52 (85%) were sensitive to NOV012 (Table 1). The cocktail was able to infect significantly more *S. aureus* strains compared to single K710 application (*p* = 0.0022). The cocktail lysed more strains compared to P68, however this was not statistically significant. Five CRS isolates were identified to be methicillin-resistant *S. aureus* (MRSA) isolates. Three of the five MRSA isolates were susceptible to K710 and all five MRSA isolates were susceptible to P68. Efficiency of plating for each isolate ranged from 0.002 to 4-fold for K710, 2.7E-06 to 6.7-fold for P68 and 4E-06 to 8-fold for NOV012.

**Table 1:** **Sensitivity of *S. aureus* isolates of CRS origin to bacteriophage lysis (efficiency of plating indicated in brackets)**.

**Pulsotype**	**K710**	**P68**	**NOV012**
A1	LWO	LWO	LWO
A2	+++ (0.07)	++ (0.006)	+++ (0.3)
A3	+ (0.004)	+ (0.0003)	+ (0.003)
B1	+++ (0.7)	+ (0.0006)	+++ (0.5)
B2	LWO	+++ (0.2)	+++ (0.2)
C1	LWO	++ (0.0002)	+ (0.0003)
C2	++ (0.2)	R	++ (0.1)
C3	+ (0.02)	R	+ (0.02)
C4	R	+ (2.7E-06)	+ (4E-06)
C5	+ (0.002)	LWO	+ (0.004)
C6	R	+ (6E-06)	+ (4E-06)
D1	LWO	R	LWO
D2	R	R	R
D3	LWO	+ (0.003)	+ (0.9)
D4	LWO	R	LWO
E	+++ (0.5)	+++ (0.26)	+++ (0.6)
F	+++ (0.02)	+++ (0.19)	+++ (0.29)
G	+ (0.007)	R	+ (0.008)
H1	LWO	LWO	LWO
H2	R	+++ (2)	+++ (3)
H3	R	R	R
H4	+++(0.58)	+++ (3)	+++ (3)
H5	+++ (0.19)	+++ (0.02)	+++ (0.53)
H6	+++ (0.3)	+++ (1.1)	+++ (2)
I1	+++ (4)	+++ (2)	+++ (5)
I2	+++ (0.3)	+++(0.03)	+++ (3)
J	+++ (0.13)	+++ (4)	+++ (6)
K	+++ (0.74)	+++ (0.04)	+++ (0.79)
L	+++	+++	+++
M	+++	LWO	+++
N1	LWO	++	++
N2	+++ (0.25)	++ (0.9)	+++ (0.6)
N3	+++ (0.1)	+++ (0.5)	+++ (3)
O1	+++ (0.5)	+++ (1.3)	+++ (0.4)
O2	+++ (0.2)	+ (0.0006)	+++ (0.5)
O3	+++ (0.14)	R	+++ (0.16)
P	LWO	LWO	LWO
Q	LWO	+++ (0.24)	+++ (0.14)
R1	R	+++ (4)	+++ (6)
R1	R	++ (0.15)	++ (0.2)
R2	+++ (0.09)	+++ (3.8)	+++ (2.7)
R3	R	+++ (5.3)	+++ (8)
R3	R	+++ (0.9)	+++ (1.1)
R4	R	+++ (2)	+++ (3)
R5	+++ (0.4)	+++ (2)	+++ (3.3)
R6	+++ (0.27)	+++ (2.7)	+++ (4)
S1	++ (0.05)	+ (0.0038)	++ (0.11)
S2	+++ (0.08)	++ (0.001)	+++ (0.4)
T1	LWO	R	LWO
T2	LWO	+++ (6.7)	+++ (5.3)
T3	+++ (0.07)	+++ (0.47)	+++ (0.27)
T3	+++ (0.04)	+++ (0.74)	+++ (0.79)
T4	+++ (0.6)	R	+++ (1.2)
T5	LWO	+++ (0.19)	+++ (0.53)
U	+++ (0.7)	++ (0.0006)	+++ (0.54)
V1	+++ (0.27)	+++ (0.03)	+++ (0.25)
V1	+++ (0.1)	+++ (0.4)	+++ (7.3)
W	LWO	R	LWO
X	+++ (0.93)	+++ (0.09)	+++ (1.2)
Y1	+++ (0.12)	+++ (0.02)	+++ (0.3)
Y2	LWO	+++ (0.15)	+++ (1.13)

*Showing lysis of S. aureus isolates distinguished on the basis of pulsed-field gel electrophoresis (“pulsotypes”). NOV012 lysed 52/61 (85%) of isolates. LWO, lysis from without; R, resistant; +, slightly sensitive; ++, moderately sensitive; +++, highly sensitive. Gray shaded isolates represent MRSA strains. All strains where obtained from The Queen Elizabeth hospital, Woodville, Australia*.

### Animal studies

#### Observations of sheep general health

During phage administration to sheep, there was no change in the general well-being of three out of four sheep in each of the control group and the phage group. One sheep in the control group and one in the phage group experienced some loss of appetite during the treatment period. Both sheep were found to have infections at the site of catheter insertion, which was thought to be the cause of appetite disruption. Antibiotic treatment during the treatment period, in accordance with the ethics committee-approved experimentation protocol, improved the appetite of both sheep. The general health of all four sheep treated with HI*p* was as expected and no change in sheep well-being was observed in this group.

#### Histology of sheep sinus mucosa

**Figure 1:**
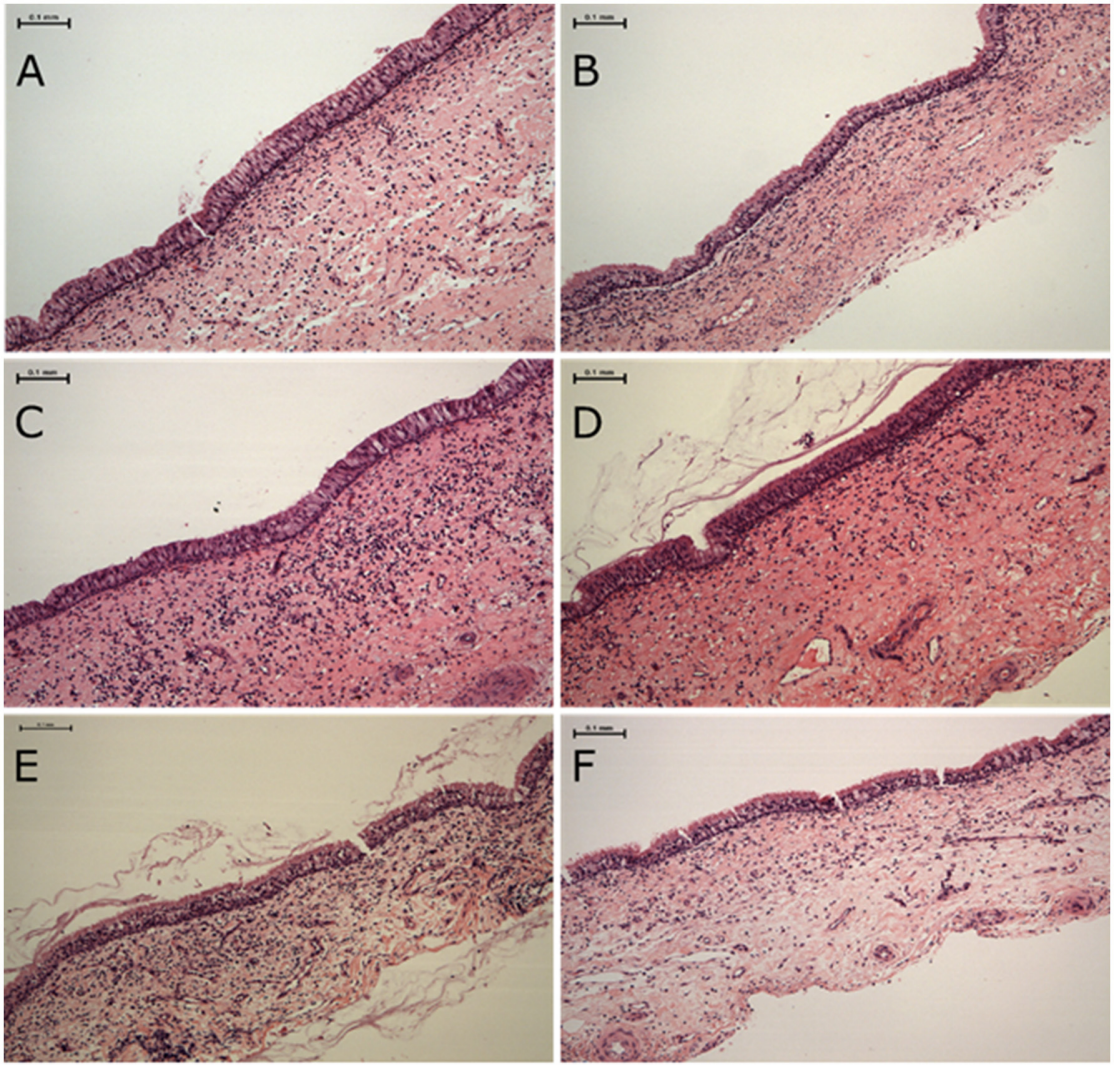
**Haematoxylin and eosin stained sheep nasal mucosa sections**. Sections are of tissues taken from animals euthanized after 20 days of treatment as below. No differences in tissue inflammation, oedema, fibrosis and the presence or absence of goblet cell hyperplasia was observed between treatment groups **(A,B)** control, **(C,D)** heat-inactivated NOV012 (Hi*p*) treatment and **(E,F)** active NOV012 treatment.

H&E stained tissue sections taken from sheep euthanized at the end of the treatment period (Figure 1) were examined for inflammation, oedema, fibrosis and the presence or absence of goblet cell hyperplasia. There were no significant differences between the groups in regard to each of these parameters (data not shown).

#### Presence and appearance of cilia of mucosa tissue

**Figure 2:**
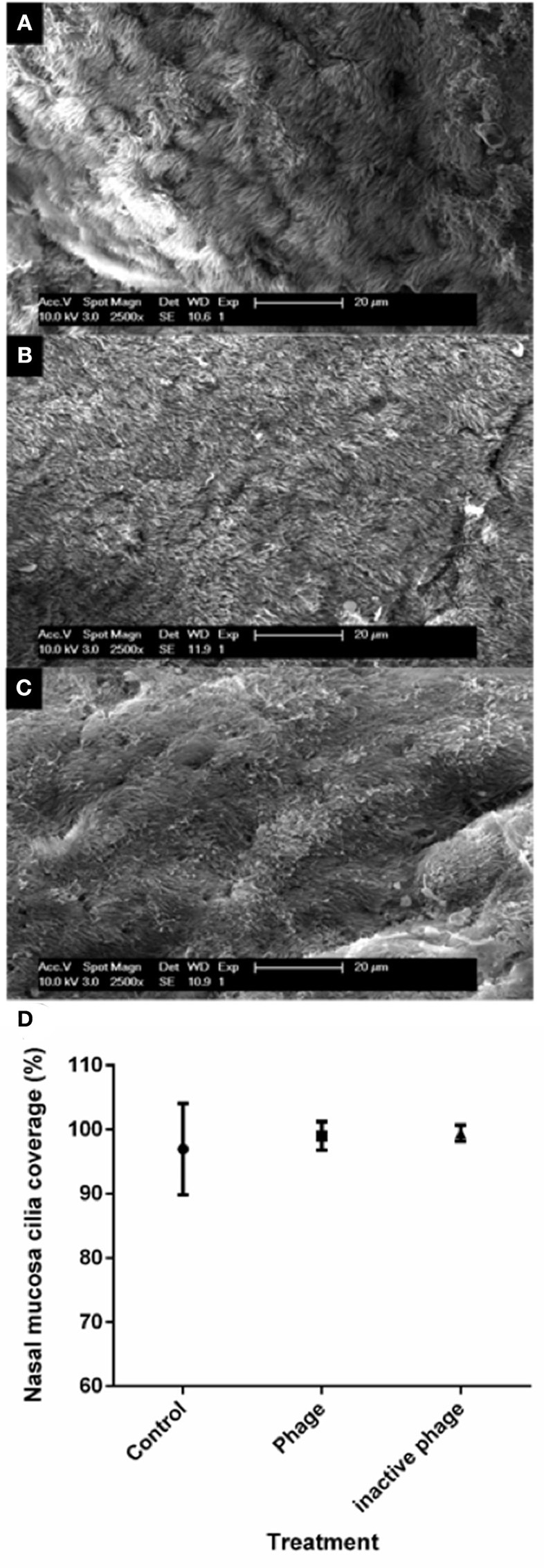
**Representative images of sheep nasal mucosa viewed using scanning electron microscopy and analysis of cilia coverage**. Cilia coverage and morphology were similar between all treatment groups including **(A)** control, **(B)** heat-inactivated NOV012 (Hi*p*) treatment, and **(C)** active NOV012 treatment. **(D)** Five images of each tissue section were captured at 2500× magnification, divided into 2 cm^2^ grid sections and each grid scored according to 1, full cilia coverage; 0.5, some cilia coverage; 0, no cilia present. This graph shows the average percentage coverage across the three treatment groups, no statistical difference was observed between the groups.

SEM was performed to allow closer inspection of sinus mucosa cilia. Cilia of all seven sinus samples harvested from HI*p* treatment were able to be visualized. The cilia of one control treated sheep sample and one phage treated sample were obscured from view by mucus, hence these samples were excluded from analysis. The appearance and coverage of the cilia was similar across the groups (Figure 2).

#### Phage detection in sheep serum

Using plaque assays, no infectious phages were detected in serum of the CONT, Hi*p*, or NOV012-treated sheep taken at any timepoint during the experiment.

## Discussion

Phage therapy has almost a 100 year history of human application for treating bacterial infections (Carlton, 1999). Accompanying this long history is an outstanding record of safety. There are numerous reports of the safety of phage therapy in animal (Soothill, 1994; Biswas et al., 2002; Wills et al., 2005; McVay et al., 2007; Hawkins et al., 2010; Park et al., 2014) and in human trials (Markoishvili et al., 2002; Bruttin and Brussow, 2005; Rhoads et al., 2009; Wright et al., 2009; Sarker et al., 2012; McCallin et al., 2013). Our previous work showed that short-term bacteriophage application is safe when applied topically to the sheep sinonasal region (Drilling et al., 2014a). This study aimed to extend this work by assessing longer-term (20 days) sinonasal phage application. This safety data is needed to support the further preclinical development of phage therapy in general and NOVO12 specifically as it is likely that phage therapy would be used in patients for an extended period of time, at least 2 weeks.

It is recognized that phage may interact with some aspects of the host immune system, and it is important to ensure they do not elicit adverse immune responses (Kaur et al., 2012). Supporting our previous work (Drilling et al., 2014a), this study shows that 20 days of topical phage therapy did not modify or damage the architecture of the sinus mucosal lining. Phage application for this extended period did not appear to increase or alter the profile of immune cells in the sinus mucosa. A limitation of this study is that only the presence of the cells was examined, whereas stimulation of the cells in relation to immune effector molecules such as cytokines was not investigated. Previous work has investigated this parameter, applying phage T4 or purified phage T4 proteins to mice and humans (Miernikiewicz et al., 2013). It was shown that such products did not stimulate the production of inflammatory related cytokines and reactive oxygen species (ROS; Miernikiewicz et al., 2013). In contrast, it has been suggested that through complex bacterial-phage-host interaction, phage can reduce ROS production (Przerwa et al., 2006; Miedzybrodzki et al., 2008). Recent research has also investigated the effect on phage infectivity when exposed to cell lines that mounted an inflammatory response to the phage. This study found that the phage still retained infectivity against bacterial cells (Khan Mirzaei et al., 2016).

It is not known whether phages can traverse the sinonasal mucosal barrier to gain access to the bloodstream. During the 20 days of treatment, no phages were detected in the bloodstream. A limitation of this study, which relied on plaque assays, is that it could not determine whether inactivated phage or phage genomes entered the bloodstream. A further limitation is that this study did not address the question of whether the sheep developed antibodies against phages. It has been shown that neutralizing antibodies may be produced following phage treatment (Kucharewicz-Krukowska and Slopek, 1987), and our future work will examine this possibility. This is of interest because it is not yet confirmed whether development of anti-phage antibodies will have a negative impact on phage therapy (Sulakvelidze et al., 2001). Recent research however has shown positive results, showing that development of antibodies may not necessarily strongly impact the clinical success of phage therapy (Zaczek et al., 2016; Lusiak-Szelachowska et al., 2017).

In addition to examining the safety profile of phage cocktail NOV012, this study also investigated the NOV012 host range in the context of CRS *S. aureus* infections. The findings of this study build on our previous work (Drilling et al., 2014a) that suggests using a cocktail of phage assisted in overcoming issues of matching phage to bacteria. We found that phage K710 was effective against 59% of the *S. aureus* strains in our panel of isolates, which was increased to 85% with addition of phage P68. Using cocktails rather than a single phage has the additional benefit of reducing the rate of generation of bacteria resistant to phage infection (Chan et al., 2013).

This work has implications beyond the treatment of CRS. For example, nasal colonization with *S. aureus* increases the risk of surgical wound infection (Perl and Roy, 1995). *S. aureus* decolonization reduces such risks (Bode et al., 2010; Chen et al., 2013), and phage have potential to achieve this. Other potential applications include topical treatment of burn wounds (Mousa, 2005) and treatment of indwelling catheter infections (Piraino, 2000). Importantly, this therapy can be broadened to target other bacterial pathogens.

## Conclusions

This work confirms that the NOV012 phage cocktail infects a broad range of *S. aureus* isolates, including a number of MRSA isolates, from CRS patients. Further, we find that longer term (20 day) topical application of the cocktail is safe for sheep sinonasal application. This safety data supports the potential for the use of phage as a topical antimicrobial treatment in CRS and will help build the profile of the product to lead to the ability to use the product in clinical trials and eventually commercially.

## Author contributions

AD executed experiments, wrote first draft. MO assisted with experiments, edited first draft. DM assisted with experiments. CJ assisted with data interpretation. PS and SV helped with experiment design, data interpretation, manuscript writing. JC helped with data interpretation. PW designed the study, manuscript writing.

### Conflict of interest statement

JC is the Chief Scientific Officer of Fixed Phage Limited and provided PW with the phage used in this study. PW is a consultant for Neilmed. The authors declare that the research was conducted in the absence of any commercial or financial relationships that could be construed as a potential conflict of interest.

## Abbreviations

NOV012: Novolytics bacteriophage cocktail
Hi*p*: Heat inactivated NOV012 bacteriophage cocktail
SEM: scanning electron microscopy
CRS: chronic rhinosinusitis.
